# Modulation of dog–owner relationship and dog social and cognitive behavior by owner temperament and dog breed group

**DOI:** 10.1038/s41598-023-41849-0

**Published:** 2023-09-07

**Authors:** Miiamaaria V. Kujala, Noora Imponen, Aino Pirkkala, Tiia Silfverberg, Tiina Parviainen, Katriina Tiira, Noona Kiuru

**Affiliations:** 1 Department of Psychology, Faculty of Education and Psychology, University of Jyväskylä, PO BOX 35, 40014 Jyväskylä, Finland; 2 Faculty of Veterinary Medicine, University of Helsinki, PO BOX 57, 00014 Helsinki, Finland; 3 SmartDOG, Pietilänkatu 5, 11130 Riihimäki, Finland

**Keywords:** Animal behaviour, Animal physiology, Social evolution, Quality of life

## Abstract

As companion dogs spend most of their lives with humans, the human–dog relationship and owner temperament may affect the dog behavior. In this study (n = 440), we investigated the relationship between the dog owner temperament (ATQ-R), owner-perceived dog–owner relationship (MDORS) and the dog behavior in three behavioral tests: the object-choice test, the unsolvable task, and the cylinder test. Dog owner temperament influenced the dog–owner relationship. Owners with high negative affectivity showed higher emotional closeness and perceived costs of their dog, whereas owners with high effortful control showed lower emotional closeness and perceived costs. Higher dog activity during the behavioral tests was also connected with owner-perceived lower emotional closeness. Furthermore, dog breed group modulated the connection between the owner temperament and dog behavior. Owner’s high negative affectivity correlated with herding dogs’ lower scores in the object choice test, while the behavior of primitive type dogs was unaffected by the owner temperament. Our results confirm that human characteristics are associated with the owner-reported dog–owner relationship, and owner temperament may have a modulatory effect on the dog social and cognitive behavior depending on the dog breed group, which should be investigated further.

## Introduction

The relationship between dogs and their owners bears similarities to the child–parent relationship^1^, and due to the increasing numbers of dogs as family pets globally, it is currently under wide interest^2,3^. Although many behavioral traits of dogs affecting the human-directed and emotional behavior are hereditary^4–7^, behavior is a multifaceted phenomenon. While hereditary factors define the prerequisites and limitations of one’s capabilities, environment fine-tunes the ways we interact and behave—and the same is true also in dogs^8,9^. Fine-tuning of the behavior is also affected by the interaction with others, either conspecific or non-conspecific. As companion dogs share much of their lives with humans, they create a large part of the daily environment for each other, and this interaction may shape dog cognition and behavior^2^ as well as those of humans^10–12^.

Certain characteristics of dog owners are associated with the dog–owner relationship. For example, higher levels of dog owner neuroticism has been associated with the dysfunctionality of dog–owner relationship as defined by dog-suffered traumas^13^; additionally, children in the family and owner’s low level of activities with the dog are associated with low emotional closeness^14^ or lower attachment^15^ of the owner with the dog. Furthermore, dog owners with high levels of psychotic tendencies have dogs belonging to breeds considered as aggressive^16^. Also, high levels of dog owner neuroticism, as well as low levels of agreeableness, emotional stability, extraversion, and conscientiousness are associated with higher levels of dog aggression- or fear-related behavior^17–19^. Furthermore, owner-filled questionnaires of the personalities of dog and the owner are associated with one another^20^ and dog owners’ personalities are further associated with their interaction with the dogs: owners with lower openness tend to show higher control of the dog, and owners with lower conscientiousness tend to show higher praising, petting and reappraising the dog^21^.

The association of owner characteristics with the dog state has also been studied on hormonal levels^22–25^. Higher owner neuroticism is associated with lower dog stress measured from the hair cortisol levels^24,25^, and both owner higher neuroticism and lower conscientiousness are associated with dog’s lower morning salivary cortisol^22^. Additionally, owner’s higher neuroticism has been associated with dog’s lower individual variability of salivary cortisol^23^. These studies suggest that despite the dog emotional reactivity has a hereditary basis, dog owners may also have a complex association with the affective states of their dog. Nevertheless, previous studies are rather diverse and studies on human effects on dog behavior have focused primarily on the dog behavior seen as problematic. To date, information about the possible association between dog owner temperament and dog–owner relationship is also largely missing. Here, we examine the connection of dog owner temperament with dog–owner relationship and dog social and cognitive behavior within the same framework.

Human temperament refers to the individual differences of human sensational and emotional reactivity, rooted in the biological-genetic prerequisites of our emotionality, as well as the cognitive modulation of the behavior^26–28^. Individual differences in temperament are already present in young infants^29^, giving rise to full-blown personality differences later in life. Temperament can be seen as the relatively stable emotional constituent of personality (e.g.^30^), and temperament factors are further connected with those of personality^31^. Nevertheless, human personality as a whole includes also other characteristics such as our goals, intentions and attitudes that can be altered in the course of life^32^. Some studies assessing the contribution of human personality in the context of human–dog interaction or dog behavior^18,24, 33^ have quantified human personality by using the Big Five Inventory (BFI^34^) or its shortened version^35^; while others^13,16^ have used Eysenck Personality Questionnaire (EPQ-R^36^). We wanted to examine whether the concept of temperament ties into the literature of dog–owner relationship, as it is widely utilized in e.g. parent–child literature^29,37–39^, but no data exists on its effects in the field of human–dog interaction. Thus, we examined the human factors under the concept of temperament, utilizing the revised Adult Temperament Questionnaire (ATQ-R^26^). ATQ-R includes four major subfactors: *extraversion* (tendency to have positive experiences and sociability), *negative affectivity* (tendency to have negative experiences such as fear or sadness), *effortful control* (ability to have cognitive control on one’s actions) and *orienting sensitivity* (tendency to notice details in the environment). These temperament dimensions correlate with the Big Five personality dimensions of extraversion (extraversion), neuroticism (negative affectivity), conscientiousness (effortful control), and openness to experience (orienting sensitivity), respectively^40,41^; see also^42^.

In addition to dog owner personality, dog behavior has been also connected to the quality of dog–owner relationship^13,43–45^. In previous studies, the quality of human–dog interaction, and in many cases, also the dog behavior, has been examined with owner-answered questionnaires. As a measure of dog–owner relationship, we selected the owner-answered questionnaire Monash Dog Owner Relationship Scale (MDORS), which quantifies three factors contributing to the relationship: *emotional closeness* between dog and the owner; *perceived costs* of the dog ownership; and the *dog–owner interaction*^46^. Previously, factors of MDORS have been associated with both dog owner demographic characteristics^14^ as well as dog characteristics such as aggression, fearfulness or physical activity^14,45,47,48^, thus it appears promising for examining also the connection of dog–owner relationship with dog owner temperament and dog social and cognitive behavior; in this study, we focus on its *emotional closeness* and *perceived costs* factors.

Previous studies have clarified the connection of dog owner personality with the indications of dog fearful, avoidant, or aggressive behavior^16–18,33^. Here, we were interested to examine the owner effect on dog behavior related to social, emotional, and cognitive functioning. Also, instead of owner-filled questionnaires to quantify the behavior of dogs, we chose to utilize direct behavioral testing with dogs to sample dog behavior—likewise to some previous research^14,33^. To provide direct information of dog behavior, we utilized well-established dog behavioral tests from a large database (smartDOG Oy), see^49^: the dog ability to use human bodily gestures as communicational cues (*object-choice test*; for review see^50^); the dog dependency of humans in the dog problem-solving strategy (*unsolvable task*/impossible task, for review, see^51^); and the dog inhibitory ability (motor inhibition) to control one’s actions as opposed to impulsive behavior (*cylinder test*^52,53^).

**Figure 1:**
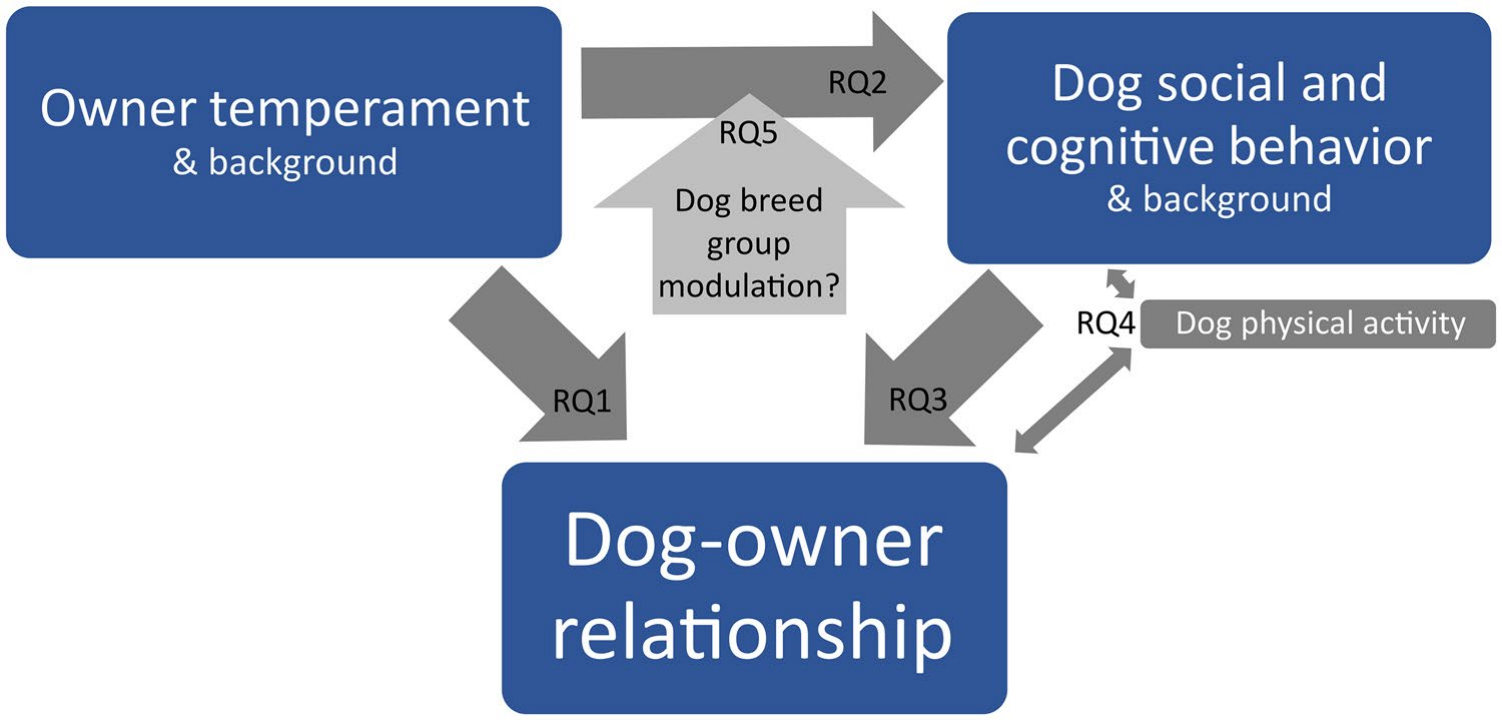
Main research questions (RQ1–RQ5). We examined the triadic connection between the dog owner temperament, dog–owner relationship, and the dog social and cognitive behavior (RQ1–3). Also dog physical activity connection with dog–owner relationship and dog behavior was examined (RQ4), and the modulation of the connection between the owner temperament and dog behavior by the dog breed group (herding dogs vs. primitive type breeds, RQ5).

The primary purpose of the current study was to investigate associations between the dog owner temperament; quality of dog–owner relationship; as well as dog social and cognitive behavior measured by the behavioral tests (Fig. 1). Our first aim (research question 1, RQ1) was to examine the extent to which the dog owner temperament (*negative affectivity, effortful control, extraversion, and orienting sensitivity*) and owner demographic background variables (number of children and number of dogs in the family) are associated with quality of dog–owner relationship (*emotional closeness* and *perceived costs*; Fig. 1). There are no prior studies examining owner temperament or personality connection with MDORS directly, but owner high levels of neuroticism have been associated with dysfunctional dog–owner relationship^13^. In a dysfunctional dog–owner relationship, personal costs of the dog ownership may be perceived as higher than in well-functioning dog–owner relationship, thus we expected high *negative affectivity* to associate with high *perceived costs*. As for the other temperament factors, prior studies only link owner personality with dog behavior, not directly to dog–owner relationship^16–18,33^, thus we have no specific expectations. Regarding the owner demographic background variables, we expected the number of children in the family to decrease owner-felt *emotional closeness* with the dog, whereas number of dogs to increase *emotional closeness* as previously^14,15^. Instead for the owner *perceived costs*, we had no expectation based on previous work.

As the dog owner personality traits have been previously associated with dog emotional behavior^18,19,33^, as well as dog stress levels^25^, our second aim was to examine whether the dog owner temperament (*negative affectivity, effortful control, extraversion,* and *orienting sensitivity*) is associated with the dog’s social and cognitive behavior (research question 2, RQ2, see Fig. 1). As there are no earlier studies on this topic, we did not have specific hypotheses on the associations.

Our third aim was to examine the association of dog–owner relationship quality with dogs’ social and cognitive behavior and dog sex (research question 3, RQ3, Fig. 1) as well as dog’s physical activity during a short behavioral test battery (research question 4, RQ4). As dog behavior is connected with human–dog interaction^13,14,44,45^, we anticipated the dog performance in the behavioral tests to be connected with the dog–owner relationship. Dog attention-seeking has been positively associated with owner attachment^44^, thus in our RQ3, the better performance of the dog in social and cognitive behavioral tasks could show a similar connection by increasing the owner *emotional closeness* with the dog. Furthermore, as low inhibitory control of the dog may predict unwanted behaviors in dogs^54^, we expected better dog inhibitory control to increase *emotional closeness*, whereas lower dog inhibitory control to increase *perceived costs*. Additionally, as dog sex affects its unwanted behavior, with female dogs having more fearfulness^8,55,56^ and dog fearfulness has been further connected to owner-felt *emotional closeness* and *perceived costs*^14^, we expect dog female sex to decrease the *emotional closeness* and increase *perceived costs*.

Regarding our RQ4, as hyperactivity and impulsivity are commonly observed unwanted canine behaviors^54,57^, dog’s high physical activity levels measured during the behavioral tests may have negative influence on the human–dog relationship, either decreasing *emotional closeness* or increasing *perceived costs*.

Finally, as dog social and cognitive behavior have strong hereditary components^5,58^, and breeds differ in their behavior in the tests utilized here^49^, we examined whether the dog breed group moderate the associations between the owner temperament and dog social behavior (research question 5, RQ5). Due to small sample sizes per breed, we investigated the effect of the breed group, similarly to some previous studies^24,25^. We focused especially in the herding dogs and primitive type dogs that have previously shown differences in responsiveness to the owner stress^24^. As the previous findings found owner neuroticism, openness and conscientiousness to associate with the dog emotional responses in the herding dogs but not in the ancient breeds^24,25^, we expected the corresponding owner temperament factors *negative affectivity*, *orienting sensitivity* and *effortful control* to affect especially the dogs in the herding group. However, as the effect of dog owner temperament to the dog social and cognitive behavior has not been studied before as such, we did not have further expectations of the connection.

## Materials and methods

### Data collection

Two types of data collection were used in the study: behavioral and activity data was collected from dogs during a dog behavioral test battery measuring dog social and cognitive behavior (for details, see^49^), and questionnaire data was collected later from their owners. Together these formed a data ensemble of the dog–owner dyads.

Behavioral and activity data of dogs were obtained within a standardized COGNITION test battery^49^ by a company that offers dog behavioral tests, SmartDOG Oy (https://www.smartdog.fi/english/), utilized in this study under a concession contract between the company and University of Jyväskylä. The dogs had participated in the behavioral test battery between 2016 and 2020.

Questionnaire data from dog owners was obtained online during 07/2020–09/2020. Dog owners were invited by email to answer the questionnaire; an invitation was also posted to the SmartDOG Oy social media profile (Facebook).

The online questionnaire was answered in due time by 671 dog owners. The data of 9 participants were removed due to technical reasons related to the questionnaires or pairing the dog–owner dyads (e.g. owner consent for data usage missing; owner answered twice; dog named by owner missing from the SmartDOG database; two owners mentioning the same dog), thus the data of 662 dog–owner dyads could be formed. After further removal of the dog–owner pairs whose data was incomplete (e.g. the dog had either not undergone the COGNITION but instead another test battery or in one case, tester mistake in writing down the test scores, rendering the test values outside the possible range), the final number of dog–owner dyads, with the complete data set with questionnaire responses from humans and behavioral data from dog participants, was 440. No data was excluded on the basis of being outliers; all the exclusions were done prior the analyses. Additionally, the dog activity measure during the behavioral testing was available from 409 dogs.

### Subjects

Dog owners were 24–74 years old (48 ± 11 years, mean ± SD; two participants did not share their age). In total, 96.4% of the owners were female, 3.2% male and 0.5% other or did not respond. 66.4% shared the household with another adult (= an individual over 18 years), 26.4% was the only adult in the household and 7.3% had two other adults in the household. Regarding children in the household, 79.1% of the participants lived without children (i.e. individuals under 18 years) in the household, 10.9% lived with a child in the household, 7.5% lived with two children, and 2.5% of the participants lived with 3 or more children in the household.

Dogs included in the study were from 6 months to 13 years old (3.6 ± 2.4 years; age was missing from three dogs). 54% of the participant dogs were female and 46% male (information missing from two dogs); 59.1% of the dogs were intact, 21.1% were castrated or sterilized (information missing from 19.8%). 38.0% of the dogs lived as the only dog in the household with their owners; 38.0% shared the household with another dog, and in 23.6% of the households, there were 2 or more dogs in addition to the dog in question. Dogs represented over 100 different breeds (mixed breed counted as one). The breed groups by the FCI classification are the following: FCI1 = sheepdogs and cattledogs; FCI2 = pinscher and schnauzer-molossoid, Swiss mountain and cattledogs; FCI3 = terriers; FCI4 = dachshunds; FCI5 = spitz and primitive types; FCI6 = scent hounds and related breeds; FCI7 = pointing dogs; FCI8 = retrievers, flushing dogs, water dogs; FCI9 = companion and toy dogs; FCI10 = sighthounds; and mixed breed (see Table 1). The list of breeds in FCI1 and FCI5 groups, which were selected for closer examination, is available in the Supplementary Table 1.

**Table 1:** Participant dogs divided by the breed groups. Values are given in mean ± SD.

Breed group	n	Age (mo)	Male/female	Intact/neutered	Object choice	Cylinder	Unsolvable task (%)		
							Human-directed	Independent	Other
FCI1	133	42 ± 28	50/54	75/29	81 ± 12	74 ± 24	47	50	3
FCI2	45	47 ± 29	15/13	19/9	80 ± 10	75 ± 24	42	54	4
FCI3	39	50 ± 27	17/12	19/11	77 ± 12	69 ± 25	46	47	7
FCI4	9	54 ± 37	3/3	5/1	73 ± 12	73 ± 19	40	58	2
FCI5	48	46 ± 39	14/29	34/9	77 ± 14	66 ± 28	50	41	9
FCI6	6	43 ± 15	4/1	5/0	83 ± 11	78 ± 12	30	68	2
FCI7	9	34 ± 20	2/3	4/1	80 ± 9	69 ± 16	51	45	4
FCI8	113	39 ± 26	36/61	81/16	80 ± 11	71 ± 25	51	45	4
FCI9	14	31 ± 18	3/11	8/6	74 ± 13	71 ± 24	45	44	11
FCI10	4	71 ± 22	3/1	3/1	81 ± 9	65 ± 24	30	53.5	16.5
mixed breed	20	40 ± 22	12/5	7/10	81 ± 14	78 ± 14	40	56	4
Total	440	43 ± 25*	159/194^†^	260/93^†^	80 ± 12	72 ± 24	46	49	5

*Information missing from three dogs. ^†^Information missing from 87 dogs.

### Owner questionnaires

The web questionnaires sampled the owner’s family background (e.g. number of children and number of dogs in the family, dog sex, age and breed) and standardized questionnaires; answering the whole questionnaire took approximately 15–30 min. The dog owner’s temperament was measured with the standardized 77-item short version of the ATQ-R^26^, validated for over 20 different languages (Finnish translation by Räikkönen-Talvitie, see^38^ and for the psychometric properties^37^) with four superfactors: *negative affectivity*, *effortful control*, *extraversion*, and *orienting sensitivity*. The superfactors consist of the following subfactors: fear, frustration, sadness and discomfort (negative affectivity); activation control, attentional control and inhibitory control (effortful control); sociability, high intensity pleasure and positive affect (extraversion); neutral perceptual sensitivity, affective perceptual sensitivity and associative sensitivity (orienting sensitivity; see Supplementary Table 2 for details). However, in this study, we were only interested in the superfactors of ATQ-R. The calculation of the superfactors was conducted as instructed in the ATQ scoring form obtained from: https://research.bowdoin.edu/rothbart-temperament-questionnaires/. The responses to the reversed items were first reverse-scored; missing scores were replaced by the mean score adding the number of Likert-scale responses for a given subject followed by dividing by the number of valid (nonmissing) responses; mean for each subfactor was calculated for each participant; and a mean was calculated across all subfactors to obtain each superfactor.

Dog–owner relationship, as perceived and evaluated by the owners themselves, was assessed with MDORS^46^ (Finnish translation in^47)^. MDORS includes three subfactors: *emotional closeness*, *perceived costs*, and *dog–owner interaction*. Each of the factor variables was calculated as the mean of the individual items within the factor. The factor reliability of MDORS was checked with Cronbach’s alpha, as it is rather new in Finland: *emotional closeness* α = 0.811; *perceived costs* α = 0.816; *dog–owner interaction* α = 0.581. As removing any single item did not improve the Cronbach’s alpha of *dog–owner interaction*, we report the results as such.

### Dog behavioral tests to measure social and cognitive behavior

The dogs participated in the behavioral tests as a part of the COGNITION test battery by smartDOG Oy; the testing situation lasted about 1.5 h and included also other tests. Tests were conducted always in the same order. In this study, we focused on the specific behavioral tests that measure the ability of the dog to utilize social communicative cues of humans (*object-choice test*); dog’s inhibitory control (*cylinder test*); and dog’s social vs. independent problem-solving strategy (henceforth referred to as *unsolvable task*). These three tasks were selected on the basis of their anticipated effect on the dog–owner relationship. The tests are described in detail by Junttila et al.^49^, and shortly summarized below.

**Figure 2:**
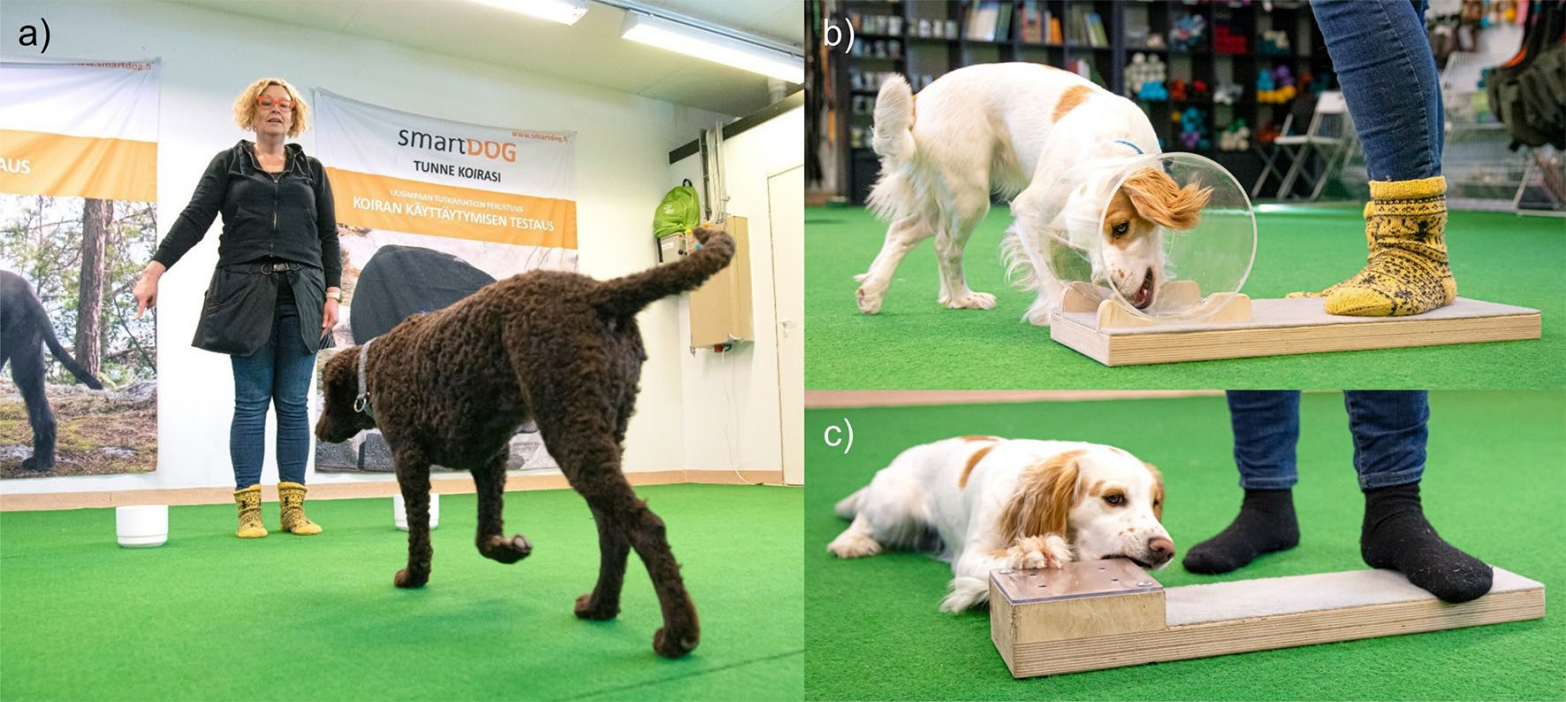
Dog behavioral tests used in this study. (**a**) One of the five variations in the object choice test, distal hand pointing, (**b**) cylinder test, (**c**) unsolvable task. All images obtained within CC by 4.0 licence from the Supplementary Information 1 of Junttila, S., Valros, A., Mäki, K. et al. Breed differences in social cognition, inhibitory control, and spatial problem-solving ability in the domestic dog (*Canis familiaris*). Sci Rep 12, 22,529 (2022). https://doi.org/10.1038/s41598-022-26991-5. Original photos by Mainossatama Oy.

In *object-choice test* (Fig. 2a), two food bowls are placed to the sides of the experimenter, about 1 m in each side, and the participant animal faces the experimenter from a few meters’ distance. First, the experimenter makes sure the animal has noticed the treat, e.g. showing the treat and letting the animal sniff it. In the warm-up, the experimenter places a treat in one of the food bowls visibly; this is repeated twice for each bowl. In the actual test, the experimenter hides the placing of the food, for example facing the other way, places the bowls to the floor and then points to the baited bowl.

There are numerous variations of the object choice test, and it has been used with a variety of different species (for review^59^). In our study, five different variations were tested (always performed in the same standardized order for each individual), each repeated in six consecutive trials, and a success rate was calculated for each variation as a percentage of correct trials (1–100%). The object choice variable was then defined as the total average of the success in five variations. First, the experimenter pointed distally at the baited bowl until the dog made a choice. The second variation was the same pointing gesture performed only for 2 s and in the third variation, the experimenter pointed to the baited bowl with a foot instead of the hand. Fourth variation was a hand pointing gesture, but performed with the contralateral hand, i.e. opposite to the baited bowl. In the final variation, repeated three times, the experimenter only gazed at the baited bowl and the dog alternatingly, without pointing with a limb. Taken together, the object choice test characterizes the ability of the animal to utilize human non-verbal gestures as a cue to find the hidden treat, and dogs outperform chimpanzees in performing the task^60^.

The *cylinder test* (Fig. 2b) is used to measure the animal’s ability to control one’s actions in order to retrieve the treat, as opposed to acting on one’s first impulses^52,53^. A cylinder open from both ends is placed in front of the animal; in the warm-up, it is rendered opaque by a cardboard sheet covering the cylinder wall. The cylinder is placed in a way that the open ends are not facing the animal but to both sides, so that a cylinder wall is nearest the animal and the animal has to retrieve the treat by either side of the cylinder. Again, a treat is visibly placed in the cylinder, and the animal may obtain the treat from either open end. After the dog retrieves the treat correctly, i.e. straight from the opening 4/5 times, the actual test phase starts. In the test phase, the cylinder is rendered transparent, allowing the animal to see the treat directly through the cylinder plexi glass. The test is repeated in ten consecutive trials, the final variable formed as a percentage of trials when the animal retrieves the treat from the open end, instead of first touching the plexi glass (always touching the glass = 0%; never touching the glass = 100%).

The *unsolvable task* (Fig. 2c) is used to clarify whether the problem-solving strategy of the dog is human-directed or independent. The dog is given a container with a treat (or, if the dog is unmotivated by a treat, a toy) inside to open. In the warm-up, the lid of the container is not fully closed, so that the dog is able to open it and retrieve the treat. In the experiment, the lid is fully closed and attached in a way that is nearly impossible for the dog to open. Thereafter, the dog is allowed to try and open the container with a treat (or a toy) inside for 2 min, and the behavior of the dog is measured as (1) a proportion of time the dog attempts to open the lid oneself (e.g. by biting or scratching); (2) the proportion of time the dog looks at the experimenter/owner, or alters gaze between humans and the box; and (3) the proportion of time the dog does something else instead of these two (e.g. moves away from the box^49^). The unsolvable task has thus three variables (*human-directed*, *independent* and *other*) that are dependent of one another, as each measure a percentage of time spent in one task instead of the other two.

### Dog physical activity

As high activity level of the dog is often considered problematic from the owner’s point of view^61^, it may also have an effect to dog–owner relationship. Dog physical activity was measured during the behavioral test battery by SmartDOG Oy, concurrently with the behavioral data. The activity was measured with a commercially available FitBark activity meter, producing 3D accelometer data, designed for dogs (FitBark inc, Kansas City, USA; https://www.fitbark.com/), selected for this purpose due its usability and availability. The output of the device are total activity points per minute, which are sampled below 1 Hz and integrated over an epoch of 1 min. The device was 4.1 × 2.8 × 1.4 cm in size and attached to the collar of the dog in the beginning of the behavioral test battery (termed “COGNITION” test battery by SmartDOG Oy). Thus, the dog physical motion was measured during the whole behavioral test battery, approx. 1.5 h.

### Statistical analysis

Data analysis was conducted in the statistical analysis software SPSS 25.0 (IBM, New York, NY, United States). Variable frequencies and distributions were examined with box plots and Q–Q plots and correlations (Spearman’s Rho) between observed variables were calculated to answer questions R1–R4, as some of the variables (dog behavioral tests and background variables) did not meet requirements for parametric statistical testing. However, as the appropriate statistical assumptions were met (e.g. the normal distribution of residuals of the dependent variables) for dog–owner relationship variables, linear regression models to predict quality of dog–owner relationship (i.e., *emotional closeness*, *perceived costs*) were constructed to further clarify RQ1, RQ3 and RQ4. In these models MDORS factors *emotional closeness* and *perceived costs* were dependent variables and ATQ-R superfactors, three dog behavioral tests (object choice, cylinder, independent problem solving in the unsolvable task) and background characteristics (number of children and number of dogs in the family, dog sex) were independent variables. Using backward elimination procedure, the final models included only statistically significant predictors.

To answer RQ5, the difference between herding and primitive type dog groups (FCI1 and FCI5) in the dog behavioral tests (object-choice test, unsolvable task and cylinder test) were assessed with independent-samples Mann–Whitney U test. Modulation of the connection between ATQ-R superfactors and dog behavioral tests by the dog breed group was further assessed Spearman’s Rho and Fisher’s Z test.

Additional analyses were also performed to examine whether people with specific temperament types more often owned dogs of a certain breed group, thus the distributions of all ATQ-R superfactors (*negative affectivity, extraversion, effortful control, orienting sensitivity*) were compared between the dog breed groups with independent-samples Kruskal–Wallis test as the overall test and pairwise comparisons conducted with independent samples Mann–Whitney U test. The possible effect of dog neutering status to the variables of interest were further checked with Spearman’s rho and independent-samples Kruskal–Wallis.

### Ethical approval

The study had a prior approval by the ethical board of the University of Jyväskylä (26.6.2020; statement #760/13.00.04.00/2020); all dog owners gave their written informed consent before participation in the study and all methods were performed in accordance with the relevant guidelines and regulations.

## Results

### Connections of owner temperament, dog–owner relationship, and dog behavioral tests

#### Correlations

**Figure 3:**
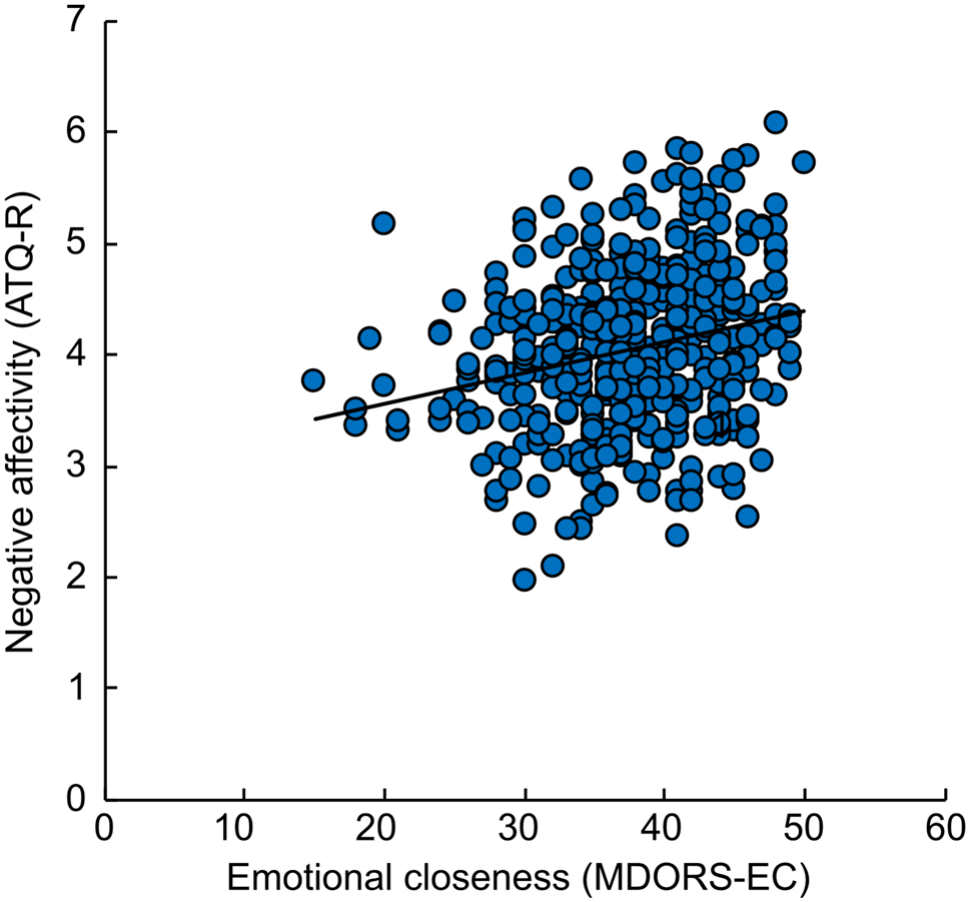
Owner negative affectivity factor of the ATQ-R (y-axis) correlated with the owner-rated emotional closeness factor of MDORS (x-axis). Higher negative affectivity was associated with higher emotional closeness.

Owner temperament measured by ATQ-R superfactors (*negative affectivity, extraversion, effortful control, orienting sensitivity*) correlated with both examined MDORS dog–owner relationship factors, *emotional closeness* (Fig. 3) and *perceived costs* (Table 2). The higher scores of the dog owner in *negative affectivity* were connected to higher scores in both *emotional closeness* and *perceived costs*, whereas higher scores in *effortful control* were connected to lower scores in both *emotional closeness* and *perceived costs*. Owner’s higher *orienting sensitivity* was only connected to higher scores in *emotional closeness*. Owner temperament factor *extraversion* was only connected to *perceived costs*: the higher *extraversion* of the owner, the lower were the *perceived costs* scores.

**Table 2:** Correlation coefficients (Spearman’s rho) between the study variables of dog–owner relationship, owner temperament, dog behavioral tests and activity.

	MDORS			ATQ-R				Dog behavioral tests + activity					
	1 EC	2 PC	3 DOI	4 Ex	5 Sen	6 Neg	7 Eff	8 Ob	9 Self	10 Soc	11 Ot	12 Cy	13 Ac
Dog–owner relationship (MDORS)													
1. Emotional closeness (EC)	1												
2. Perceived costs (PC)	− 0.166***	1											
3. Dog–owner interaction (DOI)	0.373***	− 0.157**	1										
Owner temperament (ATQ-R)													
4. Extraversion (Ex)	− 0.019	− 0.130**	0.120*	1									
5. Orienting sensitivity (Sen)	0.199***	− 0.050	0.225**	0.182***	1								
6. Negative affectivity (Neg)	0.233***	0.174***	0.090	− 0.240***	0.204***	1							
7. Effortful control (Eff)	− 0.118*	− 0.281***	0.017	− 0.143**	0.022	− 0.544***	1						
Dog behavioral tests + activity													
8. Object choice (Ob)	− 0.025	− 0.060	− 0.012	0.036	− 0.009	− 0.009	0.078	1					
9. Unsolvable task: independent% (self)	0.052	0.007	0.064	− 0.049	0.012	0.055	0.009	− 0.168***	1				
10. Unsolvable task: human-directed% (Soc)	− 0.058	− 0.026	− 0.062	0.053	− 0.035	0.090	− 0.003	0.182***	− 0.895***	1			
11. Unsolvable task: other% (Ot)	0.012	− 0.044	0.023	− 0.006	0.134**	0.092	− 0.029	− 0.096*	− 0.177***	− 0.124**	1		
12. Cylinder test: inhibitory control (Cy)	0.048	− 0.018	0.013	0.106*	− 0.066	− 0.082	− 0.034	0.083	− 0.149**	0.139**	− 0.003	1	
13. Dog activity (Ac)	− 0.098*	0.031	− 0.124*	− 0.070	− 0.019	0.074	− 0.064	− 0.050	0.151**	− 140**	− 0.024	-.073	1

*p < 0.05; **p < 0.01; ***p < 0.001; *df* for activity = 407; others *df* = 438. All tests two-tailed.

Owner *orienting sensitivity* was connected with the dog behavior in the unsolvable task and owner *extraversion* with the dog behavior in the *cylinder test*, but the MDORS factors did not correlate with dog behavioral test results (Table 2). Instead, dog physical activity during behavioral tests correlated with the MDORS: high dog activity correlated with low *emotional closeness* between the dog and the owner and low *dog–owner interaction*. Furthermore, the dog activity correlated with the dog behavior in the unsolvable task: high dog activity correlated with the dog spending more time in independent problem-solving strategy; and spending less time in human-directed problem-solving strategy.

Dog performance in the different behavioral tests (*object choice, unsolvable task, cylinder test*) significantly with each other (Table 2). Dogs who showed more human-directed behavior in the unsolvable task performed better in the object-choice task and scored higher in the cylinder task. Furthermore, the background variables of the owner were connected with MDORS factors: higher number of children correlated with higher *perceived costs* and lower *dog–owner interaction;* also higher number of dogs correlated with lower *dog–owner interaction* (Supplementary Table 3). Dog female sex was associated with higher *perceived costs*; owners of female dogs also owned more dogs. Dog neutering status had no association to the variables of interest (all *p* > 0.05).

#### Regression models

The results of regression model for *emotional closeness* (r^2^ (4) = 0.09) showed that the owner temperament (*negative affectivity,* standardized β = 0.215, *p* < 0.001 and *orienting sensitivity* β = 0.143, *p* = 0.003, respectively) and the number of children in the family (β = − 0.110, *p* = 0.017) were main factors affecting the *emotional closeness* between the dog and the owner. The higher scores of dog owner in *negative affectivity* and *orienting sensitivity* increased the *emotional closeness*, whereas higher number of children in the family decreased *emotional closeness*. Additionally, dog behavioral performance in the cylinder test remained in the final model of the *emotional closeness*, thus it could not be completely overlooked as a modulator of the *emotional closeness*, although its contribution did not reach statistical significance with two-tailed significance testing (β = 0.079, *p* = 0.087).

The results of regression model for *perceived costs* of the dog for the owner (r^2^(4) = 0.13) showed that dog owner temperament was a main factor affecting the *perceived costs* (*effortful control*, standardized β = − 0.281, *p* < 0.001 and *extraversion*;, β = − 0.102, *p* = 0.026, respectively), along with the dog sex (β = 0.148, *p* = 0.001) and number of dogs in the family (β = − 0.131, *p* = 0.004). Both owner *effortful control* and *extraversion* diminished the owner-felt *perceived costs*; also owners with more dogs in the family evaluated the *perceived costs* lower. Further, dog sex also affected the *perceived costs*, i.e. owners of male dogs evaluated the *perceived costs* as lower than owners’ of female dogs.

### Modulation of dog behavior by the dog breed group and owner temperament

**Figure 4:**
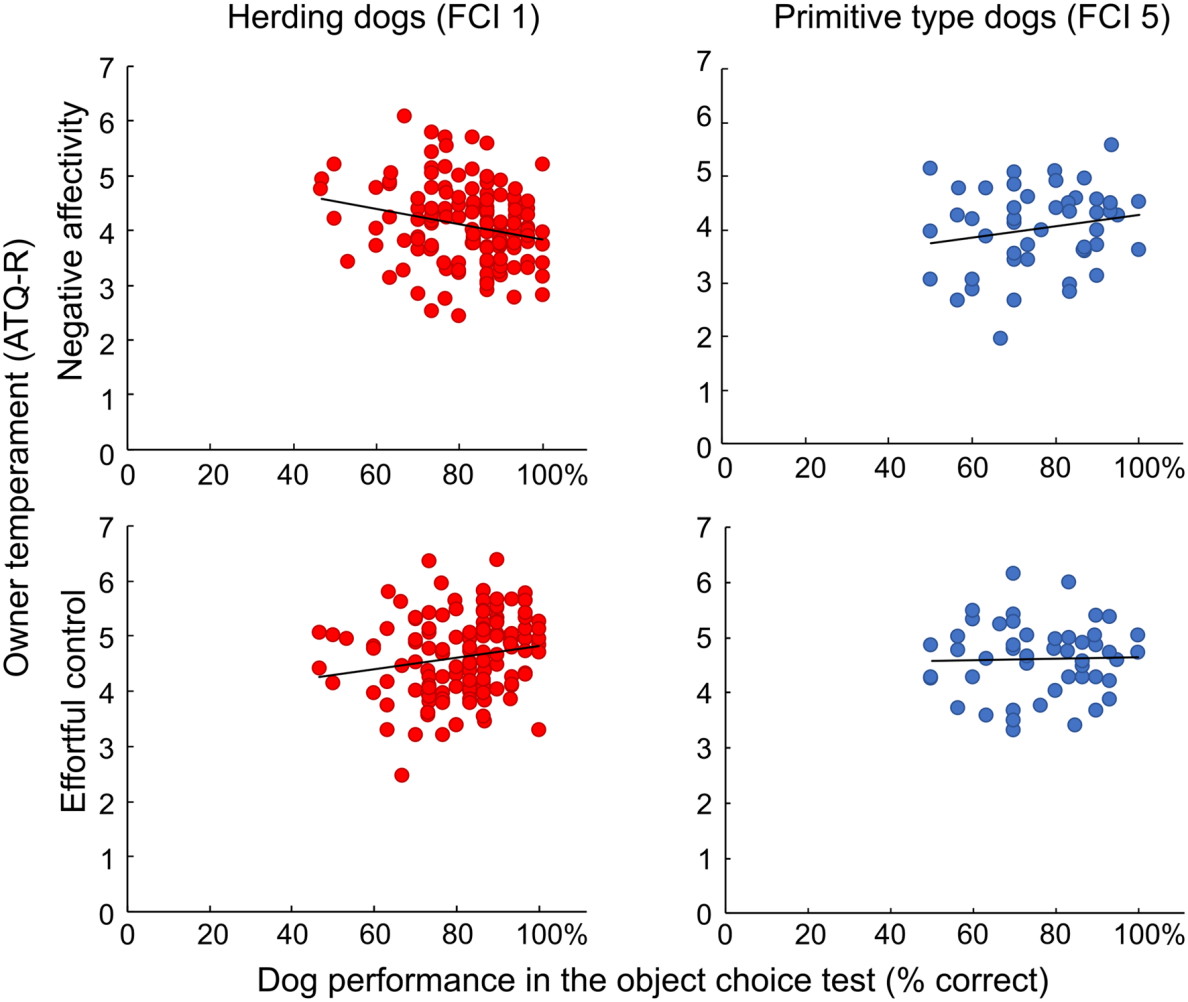
Better performance of the herding dogs (FCI1, left) in object choice test correlates with owners’ lower scores in negative affectivity and higher scores in effortful control, whereas the owner temperament correlation with the performance of the primitive type dogs (FCI5, right) was not found. Red = FCI1 group dogs, blue = FCI 5 group dogs. The difference of correlations between breed groups was significant for owner negative affectivity and dog object choice task.

Table 3 shows correlations between owner temperament and dog behavior separately for FCI1 and FCI5 breed groups. The results showed that owner temperament correlated with the performance of FCI1, but not with FCI5 dogs. Owners’ higher *orienting sensitivity* was associated with FCI1 dogs’ lower *inhibitory control* in the cylinder test. Also *object choice* performance was affected by owner temperament differently in FCI1 dogs than in FCI5 dogs: higher owner *negative affectivity* correlated with lower and *effortful control* with higher level of *object choice* performance in FCI1 dogs but not in FCI5 dogs (Fig. 4). The correlation coefficients of the owner *negative affectivity* and the dog performance in the *object choice* test significantly differed between FCI1 and FCI5 groups (Fisher’s Z = − 2.351; *p* = 0.009), whereas the correlation of the owner *effortful control* and dog *object choice* or owner *orienting sensitivity* and *cylinder test*, obtained for FCI1, did not differ between breed groups (Z = 1.304; *p* = 0.096 and Z = − 0.547; *p* = 0.292), respectively).

**Table 3:** Correlation coefficients (Spearman’s rho) and significance values of owner temperament and dog behavioral tests for the dog breed groups FCI1 and FCI5 separately.

ATQ-R	Breed group	Object choice		Cylinder test		Unsolvable task					
						Independent%		Human-directed%		Other %	
		Rho	*p*	Rho	*p*	Rho	*p*	Rho	*p*	Rho	*p*
Extraversion	FCI1	0.067	0.447	0.142	0.102	− 0.137	0.116	0.107	0.222	0.058	0.504
	FCI5	0.138	0.350	0.195	0.185	0.079	0.595	− 0.128	0.387	− 0.110	0.458
Orienting sensitivity	FCI1	− 0.038	0.667	− 0.177	0.041*	0.012	0.893	− 0.022	0.801	0.038	0.662
	FCI5	− 0.003	0.986	− 0.084	0.572	0.108	0.467	− 0.148	0.315	0.173	0.239
Negative affectivity	FCI1	− **0.216**	0.013*	− 0.161	0.064	0.006	0.942	0.003	0.973	0.047	0.592
	FCI5	**0.185**	0.209	− 0.028	0.852	0.105	0.479	− 0.242	0.098	0.190	0.197
Effortful control	FCI1	0.216	0.013*	− 0.103	0.238	0.080	0.359	− 0.087	0.321	− 0.026	0.763
	FCI5	− 0.006	0.968	0.199	0.176	− 0.091	0.539	0.029	0.844	0.012	0.935

*p < 0.05; **p < 0.01; ***p < .0.01; FCI1 *df* = 131; FCI5 *df* = 46. All tests two-tailed. Bold type indicates significantly different coefficients by Fisher’s Z, *p* < 0.01.

### Dog breed group differences in performance and owner temperament

For the performance on the behavioral tests by each dog breed group, see Table 1. The further comparison between the herding and primitive type dog groups (FCI1 and FCI5) in the dog behavioral tests showed slightly higher performance of herding dogs in the object-choice test (mean rank 95.9 vs. 77.4; Z = 2.1, *p* = 0.036; Mann–Whitney U test). Difference of FCI1 and FCI5 groups in the cylinder test could not be established (mean rank 95.3 vs. 79.2; Z = 1.84, *p* = 0.066) nor in the performance of the unsolvable task (i.e. the time the dog spent in independent or human-oriented task solving strategy or something else).

Additional analyses showed that the owner temperament factor distributions did not differ between the different FCI groups (all *p* > 0.05); similarly, i.e. none of the breed groups showed overrepresentation of a certain owner temperament type. Furthermore, the dog–owner relationship measured by MDORS factors did not differ between the FCI groups (all *p* > 0.05).

### Summary

Table 4 summarizes the findings respective to each of the RQ’s.

**Table 4:** Summary of the findings.

#	Research question (measures)	Findings
1	Dog owner temperament and owner background association with quality of dog–owner relationship (ATQ-R-MDORS)	Higher owner negative affectivity was related with higher emotional closeness and perceived costs Higher owner effortful control was related with lower emotional closeness and perceived costs Higher owner orienting sensitivity was related with higher emotional closeness Higher owner extraversion was related with lower perceived costs Higher number of children was related with higher perceived costs and lower dog–owner interaction Higher number of dogs was related with lower dog–owner interaction
2	Dog owner temperament association with the dog’s social and cognitive behavior (ATQ-R-dog behavior)	Higher owner orienting sensitivity was related with dog performance (% other than solving the task) in the unsolvable task Higher owner extraversion was related with higher dog performance in the cylinder test
3	Dog–owner relationship association with the dogs’ social and cognitive behavior and dog background (MDORS- dog behavior)	MDORS factors were not related with dog social and cognitive behavior Higher perceived costs were related with dog female sex
4	Dog–owner relationship association with the dog’s physical activity in behavioral tests (MDORS-dog activity)	Higher dog activity was related with lower emotional closeness and lower dog–owner interaction
5	Dog breed group effect on the association between the owner temperament and dog social and cognitive behavior (ATQ-R-dog behavior)	In FCI1 but not FCI5, lower owner negative affectivity and higher effortful control were related with higher dog performance in the object choice In FCI1 but not FCI5, higher owner orienting sensitivity was related with lower dog performance in the cylinder test The association between owner negative affectivity and the dog object choice test significantly differed between FCI1 and FCI5 groups

## Discussion

### Owner temperament is associated with the quality of dog–owner relationship

Previous literature suggests that human factors play a role in the dog behavior and human–dog interaction^2,13^. However, the knowledge of humane factors’ effects remains rather scattered in the previous research, concentrating on the effects of demographic and family background^14^ or human personality effects on dog behavioral variables or dog-related problems^13,16–18,33^. In this study, we aimed to provide further understanding of the associations between human temperament, owner-perceived dog–owner relationship, and dog social and cognitive behavior. The results showed that dog owner temperament is moderately strongly related with the dog–owner relationship, while it is weakly associated also with some aspects of the dog social and cognitive behavior. Furthermore, owner temperament appears to be differentially associated with the behavior of dogs belonging to herding (FCI1) and primitive (FCI5) breeds of dogs.

All temperament superfactors of ATQ-R (*negative affectivity, effortful control, extraversion, orienting sensitivity*) were connected to MDORS factors measuring dog–owner relationship (RQ1). As high levels of dog owner neuroticism have been previously associated with dysfunctionality of the dog–owner relationship^13^, we expected the high owner *negative affectivity* to be associated with high *perceived costs* of the dog ownership. As expected, dog owners with high *negative affectivity*, i.e. owners who have tendency to worry about the future and to easily experience feelings of sadness and fear^26^ had high scores in *perceived costs* of the dog–owner relationship, and they had similarly high scores in *emotional closeness*. One possible explanation for these results is that for these dog owners, dogs may be a particularly important source of emotional support in their daily lives. Hence, they view their dogs as important social supporters^62^—although simultaneously perceiving dog ownership as limiting and costly. Instead, owners’ high *effortful control*—the cognitive ability to control one’s actions despite one’s emotions^26^—appeared to function as a limiting factor. Dog owners with high *effortful control* indicated both low *emotional closeness* to their dogs and low *perceived costs* of the dog ownership. This finding is in line with previous literature showing the connection of *effortful control* to low emotional reactivity and anxiety within human relationships^63^. Owner high *extraversion*, ability to easily have positive experiences^26^, was also associated with low *perceived costs* of the dog ownership.

Human temperament factor *orienting sensitivity* measures the ability of an individual to perceive and consciously reflect small changes and signals both in the environment and within oneself, such as the bird song, texture of fabrics or the effect of the weather on one’s mood, and the creative associations of the individual^26^. In our study, *orienting sensitivity* appears important in the dog–owner relationship, as dog owner’s high *orienting sensitivity* was related with the owner’s high *emotional closeness* with one’s dog, but not with *perceived costs*. As understanding dog behavior requires the owner noticing and paying attention to the subtle signals of the dog—such as the posture, position of the ears, back and tail, as well as sounds—the factor appears important for successful dog–owner relationship.

Our results confirm the previous findings^14,15^ that higher number of children in the family decrease the *emotional closeness* as expected in our RQ1. This may be due to the dogs viewed more child-like in the absence of human children^3^. Owners may also have less time for the dog in a larger family, and the dog may be perceived less as a source of social support. Background variables also explained the *perceived costs* indicated by the owner. The owner *effortful control* and *extraversion* decreased the *perceived costs*, and owners who had more dogs perceived the costs of the dog as lower. We expected the number of dogs to increase *emotional closeness* as previously^14^, but the result of it decreasing the *perceived costs* is also in line with this. The owners of multiple dogs may be either more experienced in the dog ownership, or have found dog ownership to fit their lifestyle, thus indicating ownership of one of their dogs to be less costly for their lives; it is also possible that owners who have perceived the costs of having a companion dog high are less likely to acquire another dog.

### Associations between owner temperament and dog social and cognitive behavior

Previously, high levels of dog owner neuroticism, as well as low levels of agreeableness, emotional stability, extraversion, and conscientiousness have been associated with problematic dog behavior^13,16–18,33^. As the previous studies examined dog emotional behavior related to avoidance, fear, or aggression, whereas we examined the owner effect on dog performance in social and cognitive tasks, we did not have specific expectations of the association directions. Nevertheless, differences have been found in the sensitivity of herding vs. ancient dog breed groups to the dog owner personality factors neuroticism, openness and conscientiousness^24,25^; thus in our targeted analysis, we expected the corresponding owner temperament factors *negative affectivity, orienting sensitivity* and *effortful control* to affect especially the dogs in the herding group. Our results show that the dog owner temperament had small connection to the dog’s social and cognitive behavior (RQ2), and this connection was modulated by dog breed group (RQ5), thus we discuss these two subsequently in the following.

In this study, we found small connections of the dog owner temperament with the dog’s social and cognitive behavior (RQ2). Overall, owner *extraversion* was related with better dog inhibitory control measured by the cylinder test, and the dogs with high owner *orienting sensitivity* spent more time in the unsolvable task engaged in other activities than solving the problem. As extroverted dog owners give more often positive feedback for their dogs^33^, it is possible that the owner general tendency for positive feedback aids the dog to behave calmer in the testing situation.

The dog owner temperament effect on dog behavior was further modulated by the dog breed group (as a result to our RQ5). Split correlations revealed the herding group dogs (FCI1), but not primitive type dogs (FCI5), to be affected by the owner temperament. The owner temperament was related with the performance of the herding dogs in the *object choice* test and *cylinder test*. Owner *negative affectivity* decreased, while *effortful control* increased the herding dogs’ performance in reading human gestures in the *object choice* test. However, when formally tested only the strength of the association between owner *negative affectivity* and dog *object choice* test significantly differed between herding dogs and primitive type dogs. Higher dog owner neuroticism has been previously associated with the dysfunctionality of dog–owner relationship, manifesting in the increased number of e.g. dog injuries^13^, as well as longer delays of dogs obeying the owner commands^33^. As the sensitivity for human gestures is strongly hereditary^58^ and herding dogs perform generally better than primitive type dogs in reading human gestures^49^, herding dog breeds may be sensitive for owner *negative affectivity*—for example for negative feedback received from the owner, or even owner higher levels of stress-related cortisol levels^24,64^. Instead, the behavior of primitive type dogs that are not bred for communication are unaffected by the owner. A very recent large-scale study already showed the dog performance in these behavior tests to be modulated by the dog breed^49^. Our current study continues to examine the modulators of dog behavior and shows that in addition to the dog breed, the owner temperament also influences how well the dog utilize human gestures and the extent to which the dog can control one’s actions in an exciting situation.

### The role of dog characteristics in the owner-perceived dog–owner relationship

We also asked whether the dog social and cognitive behavior or dog sex is associated with dog–owner relationship (RQ3), and whether the dog activity during the behavioral tests was associated with these two (RQ4). Previous study found dog attention-seeking to be connected with owner attachment^44^, thus we expected the dog’s performance in the behavioral tests to associate with the owner’s experience of emotional connectedness with one’s dog (*emotional closeness*). In our study, dog–owner relationship was not affected by the dog ability to read human gestures (in the *object choice test*) or problem-solving strategy (in the *unsolvable task*), thus our expectations were not met. However, as higher dog inhibitory control in the *cylinder test*, albeit not reaching statistical significance, remained one of the explanatory variables of higher *emotional closeness* in the linear model, this suggests need for future studies. Lack of inhibitory control in dogs, i.e. impulsive behavior, is associated with behavior problems and lowered serotonin levels in dogs^54^. As we did not specifically quantify dog behavior problems in this study, it is possible that the dogs with poorer inhibitory control have more behavioral problems, which can in turn affect the dog–owner relationship^45^: this could be further assessed in forthcoming studies.

We also asked whether the dog sex affects the dog–owner relationship (RQ3). We found higher *perceived costs* indicated by owners of female dogs, which may reflect for example the effect of dog fearfulness, as females are generally more fearful than males^7,8,55^. Additionally, higher *perceived costs* for females may be explained by the type of *perceived costs* questions, which sample e.g. how much the dog limits the lives of owners^46^. Female dogs’ times of heat may be experienced as also limiting the lives of owners, as it restricts e.g. dog-related hobbies or shows.

The connection of dog activity and dog–owner relationship was examined in RQ4. The dogs using more time in independent problem-solving in the impossible task showed also higher physical activity during the whole behavioral test session and vice versa, the dogs who spent more time in human-directed problem-solving showed lower activity during the behavioral test session. This suggests that the independent dogs were very active during the whole test session. Interestingly, wolves who are known for their independent problem-solving are also shown to be more explorative (and thus might also be more active) than dogs when confronting a new environment or a novel object (see^65^). As hypothesized, higher activity level of dogs in the behavioral testing was also connected to the lower owner-reported *emotional closeness*. These results are in line with our previous study, where the *perceived costs* of the dog ownership were scored higher for dogs who oriented less toward their owners, i.e. showed more independent personality^48^. Possibly, the dog activity and independency, manifested in the social and cognitive behavioral tasks, may lower the owner-felt emotional connectedness with the dog (*emotional closeness*), whereas human-directed behavior increases the owner feeling of connectedness. This is further supported by a finding that the owner attachment to their dogs is connected with the dog attention-seeking behavior^44^.

Väätäjä et al.^47^ showed that dog owners reported higher “continuous companionship provided by the dog” (an item of the MDORS *emotional closeness* factor) when higher physical activity measures were obtained from the dog within a time period for over a week. In their study, the correlation was found for only one of the *emotional closeness* items, but even so, we found a negative connection of dog activity to *emotional closeness* factor as a whole. These two activity measurements, situation-based activity as in our behavioral test, and long-term activity as in over a week by Väätäjä et al.^47^, are likely to reflect two different things: long-term activity possibly points to the greater exercise of the dog in a long term being very dependent on the owner, whereas situation-based activity points to a greater arousal-related spontaneous movement of the dog. This is supported by the negative correlation of dog physical activity during the behavioral test and the *dog–owner interaction* factor of the MDORS: dogs showing higher physical situation-based activity had less shared activities with the owner, thus perhaps having also less possibilities for directing their energy elsewhere. An alternative explanation is that the dogs with higher situation-based activity in our study were also more independent as shown by the correlation with the impossible task, thus shared activities remaining more challenging with this kind of highly independent and active dogs.

### Limitations and future directions

Previous literature has proposed the connection of dog owner characteristics and dog behavior to be a signal of certain types of humans owning certain types of dogs^16^. In our sample, none of the owner temperament factors was over-represented within any of the different dog breed types, thus we cannot attribute a strong connection between a certain temperament of the owner and a specific type of a dog. However, it should be noted that our sample consists of a strong majority of women that have taken their dogs for the behavioral tests at their own cost, thus the sample is likely biased towards dog owners with medium to high socio-economic status. Owner gender may affect the temperament types available in the sample^66^, along with cultural background^38^; also some connections have been identified with personality and socio-economic status^67^. These factors that define our sample may result in some associations, e.g. those found by Wells and Hepper^16^ to be absent in the current study sample.

In this study, we found significant evidence for the owner temperament contribution for the dog–owner relationship as measured by the owner-answered questionnaire, while the dog social and cognitive behavior did not have a statistically significant effect on the dog–owner relationship. This affirms the critical notions of the owner-answered questionnaires to be one-sided, reflecting mostly the owner perspective of the relationship, instead of taking the dogs’ perspective into account^68,69^. Also, multiple factors may contribute to the owner side of dog–owner relationship in addition to owner temperament/personality, such as family structure, positive activities with the dog or unwanted behavior by the dog^13–15,45,70^. Future studies should investigate the role of these factors in more detail.

Although we did find support for our hypotheses; owner temperament was connected to the dog–owner interaction as indicated by the owner and dog behavior, that these connections explain only small proportions of the variance within the observed variables^71^. Dog social and cognitive behavior has a high degree of heritability^5,58^, thus the results regarding dog behavior target the part of the variation not explained by genetical differences.

## Conclusions

The mechanisms of how the owners affect dog behavior or dog–owner relationship through interaction is currently under increasing scientific scrutiny^2^. In this study, we wanted to increase the understanding of the relationship between dog owner temperament, dog–owner relationship and dog social and cognitive behavior measured with well-known behavior tests. Our results show that dog owner temperament was moderately strongly connected with dog–owner relationship (MDORS) as well as dog social and cognitive behavior, whereas the dog behavior was not connected with the dog–owner relationship. Generally, our results are in line with the previous studies showing dog owner and the demographic background contribution to the dog–owner relationship^14,70^. High activity of the dog was connected to lower *emotional closeness*, which may be further connected to potentially arising unwanted behaviors with this kind of dogs^61^. As human personality also connects to the dog behavioral problems^17–19^, these different factors—human characteristics, dog behavior, and the problems in the dog behavior and health—should be further studied within the same framework in the future.

We also found that the owner temperament effect on dog behavior was modulated by the dog breed group: dogs belonging to the herding group (FCI1) showed sensitivity for the owner temperament, whereas this association was absent in dogs belonging to the primitive type breed group (FCI5). This is in line with the recent indications of the dog breed differences in the behavioral tests^49^ as well as the different long-term synchronization of the dog and the owner in herding dogs as compared with the ancient dog breeds^24,25^. Notably, the study by Höglin et al.^24^ also found differences between the dog owner personality effects between solitary hunting dogs and ancient dog breeds, thus the effect of dog owner personality/temperament on the different dog breeds and breed groups should be studied further in the future.

Furthermore, we found a connection of dog activity, measured during the behavioral test situation, with the dog–owner relationship and the behavioral test results. Our findings show that the dogs with higher physical activity were more independent in the problem-solving, and their owners indicated lower *emotional closeness* with their dog and shared less activities with their dogs. This supports the previous findings of dog independent and vigilant personality affecting the dog–owner relationship^48^, and raises a question whether more targeted characterization and pairing of dog and owner personalities might be utilized to increase the wellbeing of both dogs and their owners in the future.

### Supplementary Information

Supplementary Tables.

## Data Availability

The data that support the findings of this study are available on reasonable request from the corresponding author. The data are not publicly available due to privacy or ethical restrictions.
