# Supplementary material for: Modulation of dog–owner relationship and dog social and cognitive behavior by owner temperament and dog breed group
Source: Sci Rep. 2023 Sep 7;13:14739. doi: 10.1038/s41598-023-41849-0 (PMC10484941; doi:10.1038/s41598-023-41849-0)
Supplement: Supplementary file 1 — Supplementary Tables. [file 41598_2023_41849_MOESM1_ESM.pdf]

Supplementary material

for “Modulation of dog-owner relationship and dog social and cognitive behavior by owner temperament and dog breed group” by Miiamaaria V. Kujala, Noora Imponen, Aino Pirkkala, Tiia Silfverberg, Tiina Parviainen, Katriina Tiira, and Noona Kiuru

Supplementary table 1. List of breeds in FCI1 and FCI5 groups.

Breed group	n	Breed
FCI1	11	Australian Kelpie
	13	Australian Shepherd
	1	Belgian Shepherd, Groenendael
	6	Belgian Shepherd, Malinois
	5	Belgian Shepherd, Tervueren
	24	Border Collie
	2	Bouvier
	1	Briard
	1	Buhund
	4	Dutch Shepherd
	1	Lancashire Heeler
	4	Mudi
	4	Bearded Collie
	3	Miniature American Shepherd
	5	Rough Collie
	2	Polski owczarek nizinny
	3	Pumi
	2	Pyrenean Sheepdog
	20	German Shepherd
	2	Schapendoes
	1	Serra de Aires Shepherd
	10	Shetland Sheepdog
	4	Smooth Collie
	3	Bohemian Shepherd
	1	White Shepherd
	1	Welsh Corgi Pembroke
FCI5	1	Alaskan Malamute
	1	Cirneco dell'Etna
	1	Icelandic Sheepdog
	1	Karelian Bear Dog
	1	Keeshond
	8	Lapponian Herder
	6	Swedish Vallhund
	3	Mittelspitz
	1	Portuguese Podengo
	1	Shiba
	1	Shikoku
	1	Siberian Husky
	19	Finnish Lapphund
	2	Finnish Spitz

Supplementary table 2. A summary of the definition and item examples of each factor in short 77-item version of the revised adult temperament questionnaire, ATQ-R (Derryberry & Rothbart 1988; Rothbart, Ahadi & Evans 2000) and Monash dog-owner relationship scale, MDORS (Dwyer et al 2006).

Questionnaire	Superfactor	Subfactor: definition	Item examples
ATQ-R[a]	Negative affectivity	Fear: Negative affect related to anticipation of distress.	Sometimes, I feel a sense of panic or terror for no apparent reason.
		Frustration: Negative affect related to interruption of ongoing tasks or goal blocking.	I find it very annoying when a store does not stock an item that I wish to buy.
		Sadness: Negative affect and lowered mood and energy related to exposure to suffering, disappointment, and object loss.	Sometimes minor events cause me to feel intense sadness.
		Discomfort: Negative affect related to sensory qualities of stimulation, including intensity, rate or complexity of visual, auditory, smell/taste, and tactile stimulation.	I find certain scratchy sounds very irritating.
	Effortful control	Activation Control: Capacity to perform an action when there is a strong tendency to avoid it.	I can keep performing a task even when I would rather not do it.
		Attentional Control: Capacity to focus attention as well as to shift attention when desired.	It is very hard for me to focus my attention when I am distressed. (reversed item)
		Inhibitory Control: Capacity to suppress inappropriate approach behavior.	I can easily resist talking out of turn, even when I'm excited and want to express an idea.
	Extraversion / Surgency	Sociability: Enjoyment derived from social interaction and being in the presence of others.	I like conversations that include several people.
		High Intensity Pleasure: Pleasure related to situations involving high stimulus intensity, rate, complexity, novelty, and incongruity.	I would probably not enjoy a fast, wild carnival ride. (reversed item)
		Positive Affect: Latency, threshold, intensity, duration, and frequency of experiencing pleasure.	Sometimes minor events cause me to feel intense happiness.
	Orienting sensitivity	Neutral Perceptual Sensitivity: Detection of slight, low intensity stimuli from both within the body and the external environment.	I'm often aware of the sounds of birds in my vicinity.
		Affective Perceptual Sensitivity: Spontaneous emotionally valenced, conscious cognition associated with low intensity stimuli.	I am often consciously aware of how the weather seems to affect my mood.
		Associative Sensitivity: Spontaneous cognitive content that is not related to standard associations with the environment.	Sometimes my mind is full of a diverse array of loosely connected thoughts and images.
MDORS[b]	Perceived emotional closeness	Owner-received social support, affectional bonding, psychological attachment, companion-	My dog provides me with constant companionship.

		ship and unconditional love from the dog	
	Perceived costs	The costs of caring for a companion dog including monetary aspects, increased responsibility, and restrictions placed on the owner because of the dog	It is annoying that I sometimes have to change my plans because of my dog.
	Dog–Owner Interaction	Activities that indicate the amount of time spent together in a relationship as well as the opportunity for shared emotional experiences and reciprocal interactions	How often do you play games with your dog?

[a] Derryberry, D. & Rothbart, M. K. Arousal, affect, and attention as components of temperament. Journal of personality and social psychology 55, 958 (1988); Rothbart, M. K., Ahadi, S. A. & Evans, D. E. Temperament and personality: origins and outcomes. Journal of personality and social psychology 78, 122 (2000); [b] Dwyer, F., Bennett, P. C. & Coleman, G. J. Development of the Monash Dog Owner Relationship Scale (MDORS). Anthrozoos 19, 243–256 (2006).

Supplementary Table 3. Correlation coefficients (Spearman's rho) between the dog-owner relationship factors (MDORS emotional closeness, perceived costs and dog-owner interaction) and the background variables (number of children and number of dogs in the family; dog gender; and the status of dog neutering). Correlation of MDORS factors is given in Table 2 thus not repeated.

	MDORS			Background variables			
	1	2	3	4	5	6	7
Dog-owner relationship							
1. Emotional Closeness	1						
2. Perceived Costs	-	1					
3. Dog-owner interaction	-	-	1				
Background variables							
4. Nr of children	-.092	.118*	-.259**	1			
5. Nr of dogs	-.004	-.085	-.144**	.023	1		
6. Dog gender[a]	-.018	.111*	-.040	.095	.123*	1	
7. Dog neutering[b]	-.060	.006	.036	-.029	-.122*	.076	1

[a] 1=male, 2=female; [b] 1=intact, 2=neutered; * p <.05; ** p < .01; *** p <.001;). All tests two-tailed.
